# Therapeutic Implications of miRNAs for Muscle-Wasting Conditions

**DOI:** 10.3390/cells10113035

**Published:** 2021-11-05

**Authors:** Laura Yedigaryan, Maurilio Sampaolesi

**Affiliations:** 1 Translational Cardiomyology Laboratory, Stem Cell Biology and Embryology, Department of Development and Regeneration, KU Leuven, 3000 Leuven, Belgium; laura.yedigaryan@kuleuven.be; 2 Histology and Medical Embryology Unit, Department of Anatomy, Histology, Forensic Medicine and Orthopedics, Sapienza University of Rome, 00185 Rome, Italy

**Keywords:** skeletal muscle regeneration, miRNAs, stem cells, epigenetics, inflammation, muscle injury, muscular dystrophies, cancer cachexia, sarcopenia, RNA, exosomes

## Abstract

MicroRNAs (miRNAs) are small, non-coding RNA molecules that are mainly involved in translational repression by binding to specific messenger RNAs. Recently, miRNAs have emerged as biomarkers, relevant for a multitude of pathophysiological conditions, and cells can selectively sort miRNAs into extracellular vesicles for paracrine and endocrine effects. In the overall context of muscle-wasting conditions, a multitude of miRNAs has been implied as being responsible for the typical dysregulation of anabolic and catabolic pathways. In general, chronic muscle disorders are associated with the main characteristic of a substantial loss in muscle mass. Muscular dystrophies (MDs) are a group of genetic diseases that cause muscle weakness and degeneration. Typically, MDs are caused by mutations in those genes responsible for upholding the integrity of muscle structure and function. Recently, the dysregulation of miRNA levels in such pathological conditions has been reported. This revelation is imperative for both MDs and other muscle-wasting conditions, such as sarcopenia and cancer cachexia. The expression levels of miRNAs have immense potential for use as potential diagnostic, prognostic and therapeutic biomarkers. Understanding the role of miRNAs in muscle-wasting conditions may lead to the development of novel strategies for the improvement of patient management.

## 1. Introduction

Skeletal muscle represents a critical feature of the human body. Loss of muscle mass is a typical characteristic of aging, also occurring in many other chronic diseases, such as cancer cachexia and muscular dystrophies [1,2]. All muscle-wasting conditions have similar functional consequences, with a resultant detrimental impact on a persons’ quality of life. As therapeutic biomarkers for muscle wasting, small non-coding RNAs, microRNAs (miRNAs), have emerged as powerful tools [3]. miRNAs post-transcriptionally alter gene expression mainly by increasing translational repression [4]. These small nucleic acids are involved in a variety of biological processes and many instances of the regulation of gene expression through miRNAs remain to be deciphered. Recent literature estimates that there are more than 2300 true human mature miRNAs, and more keep emerging [5]. In recent years, miRNAs have become largely implied in the overall regulation of skeletal muscle function. It is an emerging fact that adult stem cells [6,7] can be involved in miRNA regulation by secreting miRNA-containing exosomes, extracellular vesicles with nanoscale bilayer membranes. Indeed, exposure to specific miRNA combinations can boost the myogenic differentiation of stem cells [8,9] and skeletal muscle tumor cells [10]. The aberrant expression of miRNAs is thought to be closely associated with the progression of different muscle-wasting disorders. Their implication in both embryonic myogenesis and adult myogenesis after injury leads to the belief that these small molecules may serve as potential prognostic and therapeutic biomarkers [11,12]. Here, we review interesting findings regarding altered miRNA levels and their implications, specifically in muscle-wasting conditions.

## 2. The Biosynthesis of miRNAs

**Figure 1:**
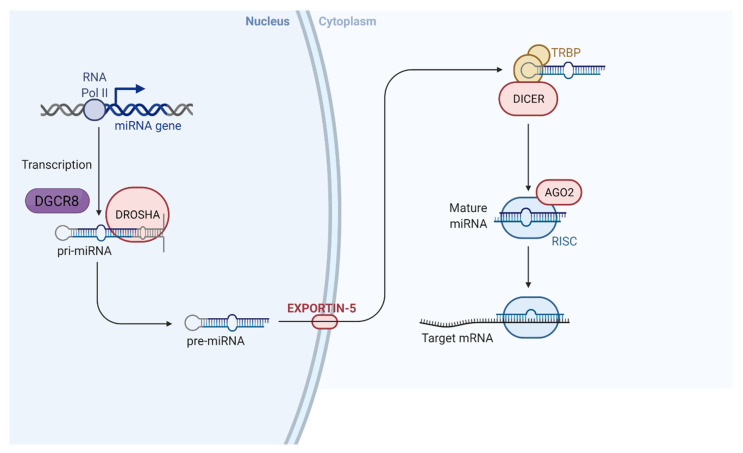
The mechanism of miRNA synthesis and action [20] (adapted from BioRender.com (accessed on 28 October 2021)). 2021 Copyright BioRender.

The canonical role of miRNAs is to bind to the 3′ untranslated region (UTR) of target messenger RNAs (mRNAs) and impose translational repression and mRNA instability [13]. The biogenesis of miRNAs involves multiple steps of cleavage, both inside the nucleus and in the cytoplasm. Initially, primary (pri)-miRNA is cleaved inside the nucleus by the microprocessor complex, composed of Drosha and Di George critical region 8 (DGCR8) [14,15] (Figure 1). Microprocessor cleavage forms precursor (pre)-miRNA. Pre-miRNA is transported to the cytoplasm by exportin-5 [16]. Consequently, Dicer cleaves pre-miRNA [17], and the resulting double-stranded miRNA is bound by Argonaute (AGO) [18]. Lastly, the miRNA-induced silencing complex (miRISC) is formed, with the guide strand remaining bound to AGO and the passenger strand removed and degraded [19]. 

Extracellularly circulating miRNAs have been identified as being either bound by proteins or encapsulated within vesicles [21]. A significant number of extracellularly circulating miRNAs have been determined to be associated with the AGO proteins [22,23,24]. This has been shown to be evident in both blood plasma, serum, and cell culture media. miRNAs have also been associated with being bound to high-density lipoproteins (HDL) or encapsulated within vesicles such as exosomes [25,26]. These nano-sized vesicles are formed from internal multivesicular bodies and can be found in body fluids such as mucus and plasma. These miRNAs are typically included in exosomes in order to circumvent degradation by freely circulating RNases. In muscles, exosomes are mainly released in an exercise-regulated fashion [27]. 

## 3. MyomiRs Regulate Crucial Events in the Muscular Niche

MyomiRs represent a group of miRNAs, expressed in a tissue-specific manner in striated muscles [28]. Thus far, seven myomiRs have been identified: miR-1-3p, -133a-3p, -133b, -206, -280a-3p, -208a-3p, and -499a-5p. miR-206 has been found to be skeletal muscle-specific, while miR-208a-3p is cardiac muscle-specific [29,30,31,32]. MyomiRs are crucial when it comes to the regulation of muscle repair, myogenesis, satellite cell activation/differentiation, and protein turnover. Other miRNAs are also involved in these processes, such as miR-23a/b clusters and miR-486-5p [31,33]. In general, the expression of myomiRs is dependent on the myogenic regulatory factors (MRFs). The MRFs represent transcription factors and cofactors that are crucial in the development and maintenance of skeletal muscle. The MRFs include myogenic factor 5 (Myf5), Myf6, Myogenin, and myoblast determination protein (MyoD) [34,35].

miR-1 and miR-206 have been shown to inhibit myogenic gene expression and to repress the expression of muscle structure and function-controlling genes [36]. Among these targets are chloride voltage-gated channel 3 (CLCN3), which is involved in fibroblast to myofibroblast transition [37]. Other genes, such as retinoic acid receptor beta (RARB) and frizzled class receptor 7 (Fzd7), are involved in cytoskeletal changes, cell growth and differentiation, and embryonic morphogenesis [38,39,40]. These miRNAs also target cell cycle progression-important genes. miR-1 and miR-206 target cyclin D1 (*CCND1*) and cyclin D2 (*CCND2*), which are positive regulators of the cyclin-dependent kinases (CDK), as well as the alpha 1 catalytic subunit of DNA polymerase (*POLA1*) [41,42,43]. Histone Deacetylase 4 (HDAC4) activity can also be regulated by miR-206 [44]. One of the main targets of miR-1 is the CACNA1C protein, which is responsible for cardiac arrhythmias [45].

miR-133a plays an important role in myoblast differentiation, partially counterbalancing the role of miR-1 [46]. miR-133a can target the 3′ UTR of the serum response factor (SRF). SRF is involved in skeletal muscle growth and maturation; therefore, the silencing of this gene leads to the maintenance of myoblasts and satellite cells in a proliferative state [46,47]. 

On the side of differentiation promotion, miR-133a targets neuronal polypyrimidine tract-building protein (nPTB) as well as Forkhead Box L2 (FOXL2) transcription factor [48,49]. miR-133a also regulates hypertrophy and actin cytoskeleton reorganization. 

As opposed to miR-133a, few pieces of evidence exist for the main targets of miR-133b. However, studies have shown that a large proportion of miR-133b targets overlaps with that of miR-133a [50,51,52,53,54]. To support this, knockout experiments for miR-1/miR-133b have not shown any serious adverse effects on regeneration, function, and the development of skeletal muscle, suggesting that miR-1/miR-133a compensate for the lack of miR-206/miR-133b [55]. 

miR-208b and miR-499a have been implicated in determining the switch between type II and type I fibers [32,56]. These miRNAs also regulate the oxidative status of skeletal muscle. 

miR-23a negatively regulates muscle atrophy by targeting two ubiquitin ligases of the ubiquitin-proteasome pathway, the muscle RING finger containing protein 1 (MURF1) and muscle atrophy F box protein (MAFBX) [57,58]. In addition to this, miR-23a suppresses the expression of SMAD Family Member 3 (SMAD3) and myostatin (MSTN) [59]. Lastly, miR-23a may act in synergy with miR-27a to downregulate both the phosphatase and tensin homolog (PTEN) and Forkhead Box O1 (FOXO1) and upregulate phosphatidylinositol 3-kinase-protein kinase B (PI3K-Akt). All in all, this miRNA opposes muscle-mass loss and contractile function. 

PTEN is a tumor-suppressor gene that has diverse functions in multiple tissues [60]. In dystrophin-deficient muscle, the increase in Pten expression levels leads to a reduction in phosphorylated Akt, both of which regulate the glucose and insulin signaling pathways in skeletal muscle [61]. 

miR-486 is mainly involved in the differentiation of satellite cells and myocytes by targeting the Paired box gene 3 (PAX3), PAX7, and MSTN [31]. By targeting PTEN, FOXO1, and MSTN, this miRNA also promotes hypertrophy and overall skeletal muscle growth. Table 1 depicts the expression pattern and prominent targets of the myomiRs. 

## 4. Implication of miRNAs in Muscle-Wasting Conditions

Muscular dystrophies (MDs) represent a group of heterogeneous inherited muscle disorders [62]. The most prominent phenotype is represented by progressive muscle weakness. In general, such diseases are difficult to treat and to understand completely. The proper regulation of muscle involves a number of undisputed events, handled by specific regulators. The overall dysregulation of such events may also lead to conditions such as cancer cachexia and sarcopenia. While cancer cachexia is due to a complex interaction between the tumor and host factors [63], sarcopenia is a muscle-wasting condition that occurs mainly with aging [64]. Recently, non-coding RNAs have taken center stage in the discussion of both pathological and homeostatic conditions. miRNAs are ~22 nucleotides long, non-coding RNAs that regulate gene expression negatively and on the post-transcriptional level [13]. 

While there are at least 34 different clinical disorders, nine major forms of MD may be identified [62,65]. These are myotonic, Duchenne, Becker, limb-girdle, facioscapulohumeral, congenital, oculopharyngeal, distal, and Emery–Dreifuss. In this review, we will mainly focus on Duchenne, Becker, myotonic, limb-girdle, and facioscapulohumeral dystrophies. 

## 5. Duchenne Muscular Dystrophy

Duchenne muscular dystrophy (DMD) is a devastating, recessive X-linked muscular disorder. DMD has an incidence of 1 in 3500–5000 male births [66,67]. 

**Figure 2:**
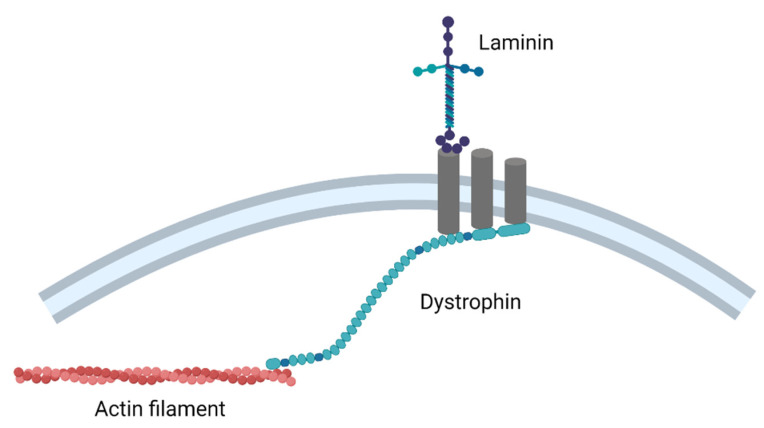
The dystrophin–glycoprotein complex (created with BioRender.com (accessed on 28 October 2021)).

DMD is caused by mutations in the dystrophin gene (*DMD*), which is responsible for the production of the muscle isoform of dystrophin. Mutations in this gene are also responsible for Becker muscular dystrophy (BMD), a milder and more manageable form of muscular dystrophy [68,69]. Dystrophin links the extracellular matrix and cytoskeletal F-actin in muscle, through the dystrophin–glycoprotein complex (Figure 2). Both frameshift and nonsense mutations can cause the premature halting of protein translation, leading to a lack of, or an unstable version of, dystrophin. 

The myomiRs represent a group of miRNAs that are involved in myogenesis. Some of these miRNAs are miR-1, miR-31, miR-133, and miR-206. These miRNAs have been found to be highly expressed in the serum of DMD patients. miR-206 is a skeletal muscle-specific miRNA that plays a crucial role in skeletal muscle tissue regeneration following trauma. 

Another muscle-specific miRNA, miR-486, is significantly downregulated in the serum of DMD patients, as well as in *mdx* mice (murine DMD models). One of the targets of miR-486 is the dedicator of cytokinesis 3 (DOCK3). The expression of this gene is upregulated in DMD patients, and it regulates the PTEN/AKT signaling pathway, specifically by decreasing the expression of PTEN. Eventually, this leads to an increase in phosphorylated AKT [70]. 

Another miRNA, miR-199a-5p, has also been found to be significantly downregulated in animal models of DMD, as well as in DMD human muscle biopsies. It has been found that miR-199a-5p is involved in the WNT signaling pathway, controlling various genes such as histone acetyltransferase GCN5 (HAG1), WNT2, and frizzled class receptor 4 (FZD4) [71]. 

The reinstatement of miR-499 has been found to reduce the severity of DMD in *mdx* mice. This miRNA is also considered as a serum biomarker in both human and golden retriever models of DMD [72,73,74]. The reported target of miR-499 is the folliculin interacting protein 1 (Fnip1), a negative regulator of AMP-activated protein kinase (AMPK). Consequently, AMPK is an activator of peroxisome proliferator-activated receptor gamma coactivator 1-alpha (PGC-1α). Therefore, the inhibition of Fnip1 in myocytes activates PGC-1α and mitochondrial functions [75]. 

AMPK is an internal sensor for ATP consumption and is a key regulator of skeletal muscle metabolism. It is generally known as a catabolic agent and is involved in autophagy and protein degradation. Additionally, AMPK suppresses anabolic processes such as protein synthesis [76]. Under pathological conditions, AMPK is critical in increasing muscle function and protecting against further damage [77]. Additionally, AMPK is necessary for macrophage skewing [78] and for enhancing the myogenesis of slow-twitch, oxidative muscle [79]. 

miR-127 targets the gene-encoding sphingosine-1-phosphate receptor 3 (S1PR3). S1PR3 is a G-protein-coupled receptor for sphingosine-1-phosphate. This miRNA is predominantly expressed in skeletal muscle. In vitro, it was found that its overexpression promotes C2C12 differentiation. Furthermore, transgenic mice overexpressing miR-127 were found to exhibit an increase in satellite cell differentiation and enhanced muscle regeneration, compared to the wild-type. In miR-127 transgenic *mdx* mice, the ubiquitous overexpression of miR-127 has been shown to promote satellite cell differentiation, thus ameliorating disease severity [80].

Another important miRNA in the context of DMD is miR-29. A decreased expression of miR-29 has been found in the muscles of *mdx* mice, correlated to the promotion of fibrosis. It has been found that blocking miR-29 in myoblasts induces the differentiation of myoblasts into myofibroblasts. This occurs due to the direct effect of miR-29 on the Akt3/nuclear factor kappa-light-chain-enhancer of activated B cells (NFkB)/Yin Yang 1 (YY1) signaling and the targeting of fibrotic genes, such as fibrillin 1 (FBN1), collagen type I alpha 1 chain (COL1A1), and collagen type III alpha 1 chain (COL3A1). The restoration of miR-29 in *mdx* mice has been shown to improve the pathology of dystrophy by promoting the regeneration of muscle tissue and decreasing fibrosis [81]. Furthermore, the coupled administration of micro-dystrophin and miR-29 has been shown to restore muscle strength in *mdx* mice [82]. 

miR-200c is significantly increased in the dystrophic muscles of *mdx* mice. The overexpression of miR-200c has been shown to inhibit myoblast differentiation. The mechanism behind this phenomenon has been reported to be the targeting of FOXO1 and endothelial nitric oxide synthase (eNOS) by miR-200c, leading to an enhancement of reactive oxygen species (ROS) production. It is likely that the increased expression of miR-200c in *mdx* mice is one of the key factors responsible for amplifying muscle wasting in this model [83]. 

miR-21 is significantly correlated with the increased expression of fibrotic genes, such as collagen type VI alpha 1 chain (COL6A) and COL1A1. The significant targets of miR-21 are PTEN and sprouty RTK signaling antagonist 1 (SPRY-1). In DMD fibroblasts, the expression of PTEN and SPRY1 is significantly downregulated, correlating with the increased expression of COL1A1 and COL6A [84]. It has been found that the disease phenotype may be improved upon the inhibition of miR-21, both in *mdx* mice and DMD fibroblasts. Morgoulis et al. have found that the synthetic preimplantation factor (sPIF) has a role in regulating miR-21 expression in DMD [85]. Overall, it was found that sPIF exerts protective effects on DMD, through the upregulation of long non-coding RNA (lncRNA) H19 and miR-675 and the downregulation of miR-21 and let-7. 

Lastly, miR-31 has been studied extensively in the context of DMD. miR-31 directly targets Myf5 and promotes satellite cell quiescence. Another important target of miR-31 is dystrophin. It has been revealed that the inhibition of miR-31 using CRSPR/Cas9 technology in induced pluripotent stem cell-derived myotubes of DMD patients can restore dystrophin [86]. 

## 6. Becker Muscular Dystrophy

BMD represents a less severe form of muscular dystrophy, unlike DMD. While severe DMD is due to the absence of dystrophin, BMD is caused by the occurrence of abnormal dystrophin. BMD may be defined through the presentation of symptoms such as myoglobinuria, muscle pain, muscle cramps, heart failure, proximal weakness, weakness localized in the quadriceps, or high concentrations of serum creatine kinase in the absence of other symptoms. The progression of BMD can be fairly variable. Interestingly, cardiomyopathy in BMD patients is more pronounced than in other dystrophinopathy patients, presumably due to the “preservation” of strength putting more pressure on the already compromised cardiac musculature [87]. 

miR-206 has been found to be significantly upregulated in the serum of BMD patients, in addition to miR-146b and miR-221 [88]. miR-206 has been inferred to play a protective role in muscular dystrophies by promoting satellite cell differentiation and fusion [89,90]. These two miRNAs play prominent roles in the inflammatory response. The upregulation of these miRNAs is also evident in other muscular dystrophy types. 

As biomarkers for distinguishing DMD from BMD, some specific miRNAs, such as miR-299-5p, miR-487b and miR-362, may be processed [91,92]. These miRNAs are only significantly upregulated in DMD. 

## 7. Myotonic Dystrophy

The most common type of muscular dystrophy in adults is myotonic dystrophy. Myotonic dystrophy is characterized by progressive myotonia, muscle weakness, and multiorgan involvement. There are two distinct types of myotonic dystrophy: myotonic dystrophy type 1 and type 2 (DM1 and DM2). DM1 is caused by nucleotide repeat expansions in the dystrophia myotonica protein kinase (DMPK), whereas DM2 is caused by an expansion in the CCHC-type zinc finger nucleic acid-binding protein (CNBP) [93,94,95,96,97,98]. Repeat expansions create a toxic mutant mRNA, subsequently interfering with the RNA-splicing machinery [99]. 

Levels of muscle-related miRNAs have been correlated with the severity and progression of DM1. Serum levels of miRs-1, -133a, -133b, and -206 have been found to be elevated in DM1 patients with acutely severe muscle wasting, compared to more stable patients [45]. Koutsoulidou et al. further elaborated on the location of the DM1-specific miRNAs [21]. This research led to the conclusion that the miRNAs are encapsulated exclusively within exosomes. 

A study done by Perfetti et al. revealed eight miRNAs that have been validated as plasma-derived biomarkers of DM1 [100]. These miRNAs are: miR-140-3p, miR-454, miR-574, miR-1, miR-133a, miR-133b, miR-206, and miR-27b. Seven of these miRNAs (miR-133a, miR-1, miR-133b, miR-140, miR-206, miR-574 and miR-454) were also found to be deregulated in the plasma of a small group of DM2 patients [100]. 

Overall, the findings in DM1 patients differ from that of DMD patients, implying that these two diseases differ quite significantly in their mechanisms of pathogenesis [45,101,102]. This may justify the differences in the release of muscle constituents within the blood of patients with these disorders. 

## 8. Limb-Girdle Muscular Dystrophy

The limb-girdle muscular dystrophies (LGMDs) represent a heterogeneous group of muscle disorders that are characterized by progressive weakness of the proximal limb-girdle muscles. There is great variability in the phenotype of LGMDs. LGMDs may be classified as either autosomal-dominant or recessive [103]. These variants are also represented by LGMD type 1 (LGMD1) and LGMD type 2 (LGMD2), respectively [104]. LGMD1 has an adult age of onset and is a rare occurrence, whereas LGMD2 cases are more frequent. Thirty percent of LGMD cases are affected by LGMD2A. Physically, scoliosis, Achilles tendon shortening, and joint contractures are the most common symptoms [105]. The progression of the disease may be defined by the perpetual loss of ambulation, respiratory insufficiency and a severely reduced lung vital capacity in the advanced stages of the disease. Cardiac involvement is rarely reported [106,107]. 

While the same involvement and deregulation of the myomiRs may be found in patients with LGMD, it has been reported that the levels of these miRNAs differ significantly from other forms of muscular dystrophy [101]. As an example, miR-1 levels in the serum of LGMD patients, compared to Facioscapulohumeral muscular dystrophy (FSHD) and BMD patients, have been found to be higher. Recently, miR-206 was revealed as a potential biomarker for patients suffering from severe cases of LGMD [108]. 

Unfortunately, there are no extensive studies available on significantly deregulated miRNAs when it comes to LGMD. The field is still open for further research. 

## 9. Facioscapulohumeral Muscular Dystrophy

FSHD represents a very common type of muscular dystrophy [109]. The pattern of disease progression involves an initial weakness of facial muscles, shoulder girdles, and upper arms, followed by weakness of the lower extremities, the trunk, and more proximal muscles [110]. Two clinically indistinguishable forms of FSHD have been reported. FSHD type 1 (FSHD1) occurs as a result of the deletion of fairly large, repeated elements on the long arm of chromosome 4q. The second form of FSHD, FSHD type 2 (FSHD2), occurs seldom and is not due to deletions [111]. 

It has been reported that comparing FSHD serum samples and age-matched controls revealed the exclusivity of 8 miRNAs that are only expressed in FSHD samples [112]. These miRNAs were miR-330, miR-331-5p, miR-516b, miR-380-3p, miR-582-5p, miR-517, miR-625 and miR-34a. Interestingly, it has previously been discovered that miR-34a is upregulated in adult biopsies that were isolated from DMD, FSHD1, LGMD1 and 2, DM2 and Miyoshi myopathy patients [92,113,114]. This suggests that miR-34a may be involved in a deregulated pathway that is common to many muscle-wasting conditions. miR-517 has been shown to be up-regulated only in adult FSHD1 biopsies and not in many of the other neuromuscular diseases studied by the authors [92]. The upregulation of miR-331 has been reported recently in FSHD primary myoblasts [115]. 

The previously mentioned miRNAs are predicted to target 632 genes. As an example, miR-582-5p, miR-516b and miR-625 target A-kinase anchoring protein 2 (AKAP2), which has mainly been found to be involved in the cyclic adenosine monophosphate (cAMP) signaling pathway [116]. A target of miR-330 and miR-331-5p, retinoic acid-induced 2 (RAI2) has been identified as being involved in development. This gene is upregulated in the side population cells of skeletal muscle. Lastly, FSHD-specific miRNAs are predicted to downregulate certain genes of the tripartite motif (TRIM) family: TRIM2, TRIM6, TRIM3, TRIM34 and TRIM41. The TRIM family has been implicated in having significant roles in cell differentiation, apoptosis, signaling pathways and transcriptional regulation [117]. The implied mechanism of miRNAs, when upregulated in FSHD fetal muscle, is the overall interference of biological processes.

## 10. Cancer Cachexia

Cachexia represents a multifaceted condition that is characterized by skeletal muscle atrophy, progressive bodyweight loss, and attenuated muscle strength. The aspect of muscle atrophy is a very serious cornerstone, associated with poor quality of life, physical disability, and a poor survival rate. Patients most susceptible to cachexia are mainly those with certain cancers and other diseases in the advanced stages, such as heart failure, chronic kidney disease (CKD), and chronic obstructive pulmonary disease patients. Unfortunately, patients with cachexia have difficulty in tolerating and/or responding to interventions such as radiation and chemotherapy for cancer cachexia [118]. 

Dysregulation of miRNA levels is a common occurrence in all subsets of cancer cachexia. 

In sequencing studies of the tibialis anterior muscle, for mice that developed cachexia in association with Lewis lung carcinoma, nine miRNAs were found to be dysregulated: miR-147-3p, miR-1933-3p, miR-299a-3p, miR-511-3p, miR-3473d, miR-223-3p, miR-431-5p, miR-665-3p and miR-205-3p [119]. These miRNAs are associated with crucial cellular processes, such as cell-to-cell communication, development, growth, and inflammatory response, to name a few. Of the miRNAs identified, the most notable were miR-147 and miR-205. Previously, it was found that miR-147 ectopic expression resulted in a decreased activation of the Akt/mammalian target of rapamycin (mTOR) in breast cancer cell lines [120]. This suggests the potential role of cancer cachexia-induced upregulation of miR-147 in inhibiting protein anabolism in muscle cells. In a similar manner, miR-205 has been shown to downregulate the CAMP responsive element binding protein 1 (CREB1) [121], leading to growth suppression in colorectal cancer cells, as well as cell proliferation control through an interaction with PTEN [122], a growth repressor, in nasopharyngeal cancer. In adult skeletal muscle, miR-205 may play a role in proteasomal degradation, thereby altering muscle proteostasis in a negative manner. Specifically, miR-205 was predicted to downregulate the MYC proto-oncogene, BHLH transcription factor (MYC), thus leading to the inhibition of Akt. Furthermore, this would inactivate the downstream initiation factor eukaryotic translation initiation factor 4E (eIF4E). In parallel, FoxO3 would be activated, leading to the transcription of pro-atrophic E3-ubiquitin ligases. Pro-inflammatory molecules have also been predicted to be upregulated as a result of miR-205 upregulation. 

Next-generation sequencing has also revealed an array of differentially expressed miRNAs that are found in skeletal muscle tissues from cachexic pancreatic and colorectal cancer patients [123], these miRNAs being: miR-3184-3p, miR-423-5p, let-7d-3p, miR-1296-5p, miR-345-5p, miR-532-5p, miR-423-3p and miR-199a-3p. Targets of miR-3184-3p, miR-1296-5p, and let-7d-3p were identified. These targets play crucial roles in inflammation, innate immune response, myogenesis, adipogenesis, and signal transduction pathways. Differentially expressed miRNAs have also been evaluated in the muscle biopsies of cachectic patients with non-small lung cancer [124]. miR-424-5p, miR-424-3p and miR-450a were significantly upregulated, while miR-451a and miR-144-5p were downregulated, compared to healthy controls.

Overall, muscle protein degradation in cancer cachexia is due to the ubiquitin-proteasome system, induced through the activation of the atrogin-1/F-box only protein 32 (MAFbx) and Muscle RING finger 1 (MurF-1) [125]. Narasimhan et al. analyzed miRNAs taken from the rectus abdominus muscle biopsies of cachectic and non-cachectic patients [123]. Eight miRNAs that were upregulated in cancer cachexia patients were implicated in myogenesis, muscle metabolism and inflammation. The miRNAs were: hsa-let-7d-3p, hsa-miR-345-5p, hsa-miR-423-5p, hsa-miR-532-5p, hsa-miR-1296-5p, hsa-miR-3184-3p, hsa-miR-423-3p and hsa-miR-199a-3p. The authors showed that let-7d-3p affects muscle cell proliferation and myogenic differentiation, through the downregulation of the transferrin receptor pathway. miR-345-5p was implicated in the Akt and mTOR pathways, reducing the ability to synthesize protein. Furthermore, it was found that miR-423-5p and miR-3184-3p downregulated genes that are involved in lipid biosynthesis. Additionally, miR-532-5p targets sulfatase (SULF), which is related to the bone morphogenic protein (BMP) signaling pathway, affecting somite development. Overall, it was found that miR-532-5p may also target certain genes crucial in appetite regulation.

## 11. Sarcopenia

Sarcopenia refers to a muscle-wasting condition occurring during normal aging [126]. Patients suffering from sarcopenia experience a reduction in their quality of life as a result of their difficulty/inability to mobilize and have a higher risk of morbidity and mortality. 

Sarcopenia may be caused by multiple factors, such as the increased fibrosis of muscle, chronic inflammation, an increased number of reactive oxidative species, etc. [127]. These factors have been scrutinized as being tightly controlled by multiple signaling pathways, such as the transforming growth factor-beta (TGF-B) signaling pathway [128,129]. 

The factors disclosed previously may result in mitochondrial dysfunction, loss of muscle protein homeostasis, and a reduced number and function of satellite cells. In addition to this, the loss of alpha motor neurons and negative alterations to the neuromuscular junctions contribute to the atrophy of muscles and the shift from type II muscle fibers to type I fibers [7,130,131]. 

Unfortunately, other than improving nutrition and increasing exercise, there is no safe and proven intervention method for sarcopenia patients. Effective treatments are still undisclosed as a result of the multi-component aspect of this disorder. Additionally, specific biomarkers are also lacking. Therefore, and as a result of the development of high-throughput sequencing technology, a large number of miRNAs have been completely identified and associated with sarcopenia. 

miRNAs that are involved in the sarcopenic phenotype may be divided into specific networks in relation to the pathophysiology of the disease. These factors include involvement in satellite cell number and function, protein homeostasis, a shift in muscle fiber type, reactive oxygen species production, neurodegeneration, and fat infiltration [131]. 

There is a significant dysregulation of the myomiRs in sarcopenic muscle. miRNAs that are involved in the regulation of satellite cell quiescence and activation appear distorted in function. Some miRNAs said to be involved in the pathogenesis of sarcopenia include miR-125b, miR-223 and miR-199-3p. miR-125b and miR-223 act through their target, insulin-like growth factor (IGF)-2, while miR-199-3p targets IGF-1, regulating the PI3K/AKT/mTOR pathway and ultimately blunting the rates of protein synthesis [132,133,134]. Another miRNA, miR-487b-3p may inhibit protein synthesis by targeting insulin receptor substrate 1 (IRS1) [135]. FoxO1 and PTEN are targets of miR-486, regulating protein degradation through the inactivation of skeletal muscle atrophy signaling [136]. miRNAs have also been implied in the transformation of muscle fiber types. For example, miR-208b and miR-499 play vital roles in inhibiting fast myofiber-specific genes and activating slow myofiber-specific genes [32,137]. miR-1 and miR-133a are implicated in skeletal muscle mitochondrial dynamics [131,138]. 

miR-206 has been demonstrated to be a key regulator of signals between the motor neurons and neuromuscular junctions (NMJs) [139]. Not only miR-206 but also miR-375 [140], miR-146a [141], miR-23a [142], and miR-234 [143], have been denoted in neural (in)stability. For example, miR-234 has been found to increase resistance to aldicarb, an acetylcholinesterase inhibitor [143]. This suggests that dysregulation in miR-234 levels may lead to NMJ dysfunction. 

Muscle atrophy, caused by malnutrition and aging, instigates fibro-adipogenic progenitors (FAPS) to activate uncontrollably and differentiate into adipocytes and fibroblasts, leading to fat and scar tissue infiltration [144,145]. This inevitably limits proper muscle repair and regeneration. The myomiRs are heavily implied in the regulation of the FAP functional phenotype. miR-133 and miR-499 regulate brown adipocyte differentiation [146,147]. Lastly, miR-143 is said to promote the synthesis of triglycerides in both humans and rodents [148]. 

**Figure 3:**
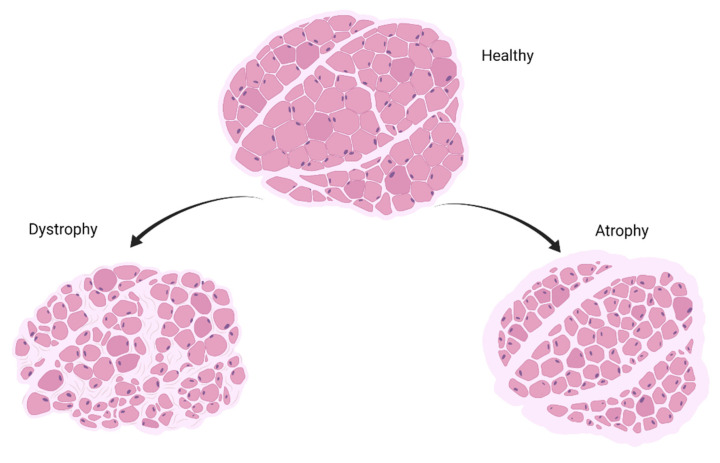
Muscle cross-sections of three conditions (healthy, dystrophic, and atrophic muscle) (created with BioRender.com (accessed on 28 October 2021)).

The general condition of healthy, dystrophic, and atrophic muscle tissues can be seen in Figure 3. Dystrophic tissue is represented through excessive fat and fibrotic tissue deposition, in addition to fibers of reduced diameter. Atrophy is represented through muscle fibers of reduced diameter, sometimes surrounded by clear spaces. 

## 12. Therapeutic Options

**Figure 4:**
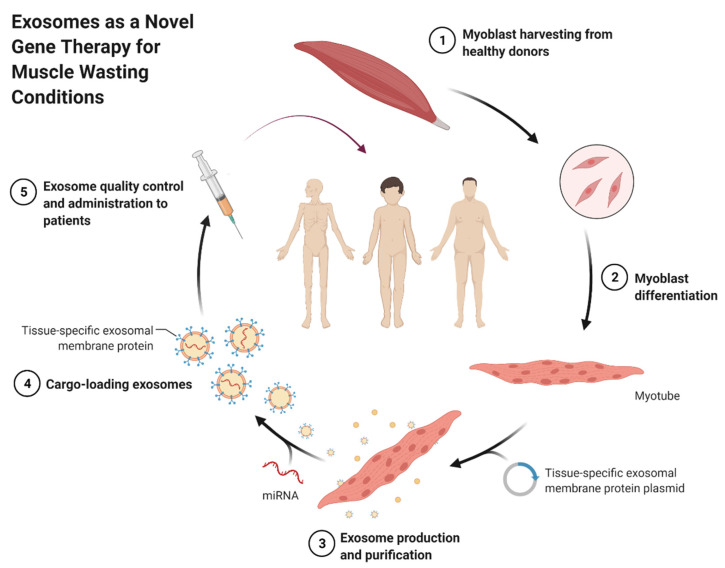
Exosomal gene therapy for muscle-wasting conditions [158] (adapted from BioRender.com (accessed on 28 October 2021)). 2021 Copyright BioRender.

Currently, most symptomatic treatments for muscle-wasting conditions, such as sarcopenia and cachexia, include orexigenic drugs, anabolic agents, and resistance training [149]. On the other hand, treatment options for muscular dystrophies involve a multitude of therapies, such as respiratory therapy, physical therapy, and speech therapy, in addition to surgical interventions and drug therapy. Recently, a potent human antibody inhibitor to MSTN has been shown to be efficacious when delivered to adult immunodeficient mice, causing an enhancement in muscle hypertrophy [150]. Unfortunately, there are still challenges when it comes to human clinical trials [151]. While many of the aforementioned options have the potential to ameliorate these conditions, there is still a very great demand for further investigation and options that can be ascertained clinically. Circulating miRNAs are proving to be capable and novel biomarkers of muscle wasting [152,153]. In addition to being stable in biofluids, miRNAs also exude limited immune reactivity; therefore, they represent excellent therapeutic candidates. miRNA therapies are still being extensively studied, and encouraging results are becoming prominent for some challenging diseases [154,155,156,157]. The main challenge for the establishment of miRNA therapy is the complete identification of miRNA targets and their potential effects. Ensuring the specificity of targets, and circumventing any toxic and off-target effects, still remain a challenging hurdle to overcome. Research in the field of miRNA usage for ameliorating muscle-wasting conditions is unfortunately still in its infancy, compared to diseases such as cancer. Much remains to be unraveled about the use of specific miRNAs and their method of delivery in muscle tissue. There are great prospects when it comes to the use of exosomes as delivery vehicles for miRNA therapy (Figure 4). It is still an open question whether the ideal source of exosomes to treat skeletal muscle-wasting conditions should be healthy donor muscle cells, stem cells or, eventually, synthetic nanoparticles, as used for COVID-19 vaccinations.

There will certainly be challenges ahead regarding pursuing miRNA therapy for muscle-wasting conditions; however, if these obstacles can be overcome, miRNA therapy may represent a powerful beacon of hope for patients suffering from these conditions. 

## 13. Conclusions

miRNAs represent a means of skeletal muscle regulation. Alterations in the expression levels of miRNAs have been demonstrated in different types of muscular dystrophies as well as in atrophic conditions, such as cancer cachexia and sarcopenia. The myogenic miRNAs (myomiRs) are particularly amenable to drastic expression pattern modifications. Studies indicate that myogenic miRNAs play a fundamental role in the pathogenesis of these conditions, hence representing potential biomarkers, as well as novel therapeutic targets. Exosome therapy, in particular, represents a potentially innovative approach to treating such conditions.

## Figures and Tables

**Table 1:** MyomiRs, their expression patterns and prominent targets.

MyomiR	Expression Pattern	Prominent Targets
miR-1-1	Skeletal muscle and heart	PAX3/7, POLA1, CCDN1/2, YY1, CX43, HDAC4, MEOX2, RARB, BAF47, BAF60A, FZD7, CNN3, SFRP1, NOTCH3, HAND2, DII-1, HES1, FRS2, myocardin
miR-1-2	Skeletal muscle and heart	-
miR-133a-1	Skeletal muscle and heart	FGFR1, PP2AC, CCN1, RUNX2, BAF60B, PRDM16, SRF, nPTB, IGF-1R, UCP2, FOXL2, FGFR1, PP2AC, ESFR, SNAI1, cyclin D2, SP1
miR-133a-2	Skeletal muscle and heart	-
miR-206	Skeletal muscle (Type I fibers)	PAX3/7, POLA1, CCDN1/2, YY1, CX43, HDAC4, MEOX2, RARB, BAF47, BAF60A, FZD7, UTM, FSTL1, nPTB
miR-208a	Skeletal muscle (mass regulator), heart	MSTN, MYH7, MYH7B, THRAP1
miR-208b	Skeletal muscle (type I fibers), heart (low expression)	SOX6, MYH6
miR-486	Skeletal muscle and heart	PAX7, PTEN, FOXO1A
miR-499	Skeletal muscle (type I fibers), heart	SOX6, MEF2C
